# Depression in Patients with Cardiovascular Disease

**DOI:** 10.1155/2012/794762

**Published:** 2012-07-05

**Authors:** Dimos Mastrogiannis, Gregory Giamouzis, Efthimios Dardiotis, George Karayannis, Artemis Chroub-Papavaiou, Dimitra Kremeti, Kyriakos Spiliopoulos, Panagiotis Georgoulias, Stelios Koutsias, Konstantinos Bonotis, Marianna Mantzorou, John Skoularigis, Georgios M. Hadjigeorgiou, Javed Butler, Filippos Triposkiadis

**Affiliations:** ^1^TEI of Lamia, Lamia, Greece; ^2^Department of Cardiology, Larissa University Hospital, P.O. Box 1425, 411 10 Larissa, Greece; ^3^Department of Neurology, Larissa University Hospital, 411 10 Larissa, Greece; ^4^Department of Cardiovascular and Thoracic Surgery, Larissa University Hospital, 411 10 Larissa, Greece; ^5^Department of Nuclear Medicine, Larissa University Hospital, 411 10 Larissa, Greece; ^6^Department of Psychiatry, Larissa University Hospital, 411 10 Larissa, Greece; ^7^Department of Vascular Surgery, Larissa University Hospital, 411 10 Larissa, Greece; ^8^Nursing Department B**ʼ**, TEI of Athens, Athens, Greece; ^9^Division of Cardiology, Emory University Hospital, Atlanta, GA, USA

## Abstract

It has been widely suggested that depression negatively affects patients with cardiovascular disease. There are several pathophysiological mechanisms as well as behavioral processes linking depression and cardiac events. Improvements in nursing and medical care have prolonged survival of this patient population; however, this beneficial outcome has led to increased prevalence of depression. Since mortality rates in chronic heart failure patients remain extremely high, it might be as equally important to screen for depression and there are several valid and reliable screening tools that healthcare personnel could easily employ to identify patients at greater risk. Consultation should be provided by a multidisciplinary team, consisting of cardiologists, psychiatrists, and hospital or community nurses so as to carefully plan, execute, and evaluate medical intervention and implement lifestyle changes. We aim to systematically review the existing knowledge regarding current definitions, prognostic implications, pathophysiological mechanisms, and current and future treatment options in patients with depression and cardiovascular disease, specifically those with heart failure.

## 1. Introduction

During the past decades, researchers have documented a significant negative impact of depression on various outcomes in patients with cardiovascular disease (CVD). Apart from the cardiac disease itself it is not uncommon that these patients experience the burden of another condition such as depression, often contributing to the worsening of their somatic illness, thus, entrapping them in a possible vicious circle.

Minor forms of depression have been reported in 20% of patients after an acute myocardial infarction (AMI), while rates of major depression vary between 16% and 45% of patients after an AMI [1, 2]. Approximately two-fifths of patients with angina suffer from depression [3]. The incidence increases in patients following coronary artery bypass graft surgery [4–6]. Depressive symptoms appear to be associated with severe functional limitation in patients with chronic heart failure (CHF) after discharge [7], and functional limitation may be viewed as a behavioural factor affecting the progression of the disease. However, it is not clear whether there is a distinct link between depression and cardiac events as well as their chronological sequence. Some authors have proposed that depression may be both an antecedent and a consequence in patients with CHF [8–10]. 

Huffman et al.reported that depression is associated with a 60% increase in the onset of cardiac diseases in a population of healthy men and women [11]. In another study, Carney et al. found that patients with major depressive disorder (MDD) who had undergone cardiac catheterization were more likely to have acute cardiovascular or ischemic events compared to non-MDD patients (77.7% versus 34.9%, relative risk 2.2, *P* < 0.02) [1]. Finally, a clinical diagnosis of depression has been found to be a strong predictor of death in patients after a recent myocardial infarction (MI) [12]. 

We aim to systematically review the existing knowledge regarding current definitions, prognostic implications, pathophysiological mechanisms, and current and future treatment options in patients with depression and cardiovascular disease.

## 2. Unveiling Depression

### 2.1. Definition

According to the fourth revised edition of the Diagnostic and Statistical Manual of Mental Disorders (DSM-IV-TR) [13], there are several criteria that need to be met in order to diagnose a single episode of major depression. The patient has to present with either depressed mood or loss of interest or pleasure during an at least two-week period along with another four additionalsymptoms: fatigue or loss of energy;diminished ability to think/concentrate; insomnia or hypersomnia; feelings of worthlessness, excessive or inappropriate guilt; recurrent thoughts of death or suicidal ideation; psychomotor agitation or retardation; significant weight loss or weight gain. The above should not be due to substance use or a medical condition. Significant distress or impairment should be present and should not be accompanied by a bereavement. In Table 1 all DSM-IV-TR criteria are presented along with the corresponding criteria according to the 10th revision of the International Statistical Classification of Diseases and Related Health Problems (ICD-10) [14]. However, the severity of depression varies and there are also other similar forms such as atypical depression or dysthymia that should not be overlooked.

It should also be noted that caution must be exercised in this particular population, since symptoms such as fatigue, dyspnea, exhaustion, and disturbed sleep occur commonly and may be confused for depressive symptoms.

### 2.2. Diagnosing Depression

In caseswhendepression is suspected in patients with cardiovascular diseases, a referral to a psychiatric clinician should be made. It could be argued that the formal diagnosis of depression should be performed by appropriately trained clinicians; nevertheless, it is widely apparent in the literature that many patients are dealt with as depressed based on numerous diagnostic tools that are utilized by nonpsychiatric clinicians.

Several instruments have been constructed and have been broadlyused: the Beck Depression Inventory (BDI) and BDI-II [15, 16], the Mental Health Diagnostic Interview Schedule [17], the Symptom CheckList-90 Revised (SCL-90-R) [18], the Hospital Anxiety and Depression Scale (HADS) [19], the Schedule for Clinical Assessment in Neuropsychiatry [20], the Medical Outcome Study-Depression (MOS-D) [21], the Zung Self-Rating Depression Scale [22], the Geriatric Depression Scale (GDS) [23], the Centre for Epidemiologic Studies-Depression Scale (CES-D) [24], the General Health Questionnaire (GHQ) [25], the Hamilton Rating Scale for Depression (HAM-D) [26], the Montgomery and Asberg Depression Rating Scale (MADRS) [27], and the Cardiac Depression Scale (CDS) [28]. 

These instruments largely address the need to assess whether patients suffer from depression when conducting epidemiological studies. Since it would be difficult, time consuming, and costly to conduct structured clinical interviews, especially in large scale trials, the above scales and questionnaires are being used successfully in everyday clinical practice.

## 3. Physiologic Mechanisms and Elements of**** Pathophysiology

Several potential physiological mechanisms linking depression and adverse cardiac events have been proposed and they are eloquently presented in the literature [11, 29]. Among these mechanisms there are increased platelet activity and aggregation, inflammation, heart rhythm disturbances, elevated levels of catecholamines, and endothelial dysfunction.

### 3.1. Platelet Activity and Aggregation

Serotonin seems to play a role in clot formation, since it can bind to 5-hydroxytryptamine (5-HT) receptors on platelets, causing the release of precoagulant factors, thus promoting platelet aggregation [30]. Also, Laghrissi-Thode et al. demonstrated that increased platelet activation is present in depressed patients regardless of the presence of coronary heart disease (CHD) [31]. It is also known that activated platelets enhance progression of atherosclerotic plaques and depressed patients have been shown to have increased levels of platelet induced chemotactic and mitogenic factors, as discussed in detail by Nemeroff et al. [32, 33].

### 3.2. Inflammation

Depression has been found to be associated with elevated levels of interleukin-1 (IL-1), IL-6, and tumor necrosis factor-*α* (TNF-*α*). These proinflammatory cytokines play a key role in the process of atherosclerosis [34]. Also, it has been shown that levels of C-reactive protein (CRP), IL-6, and TNF-*α* were higher in patients with coronary artery disease (CAD) and were linked to an increased risk of adverse cardiac events and mortality [35–37]. These findings have been supported by another meta-analysis, which concluded that this pattern existed in both clinical and community-based samples as well as in the studies which used either clinical interviews or self-report measures of depression [38]. Also, cytokines may also influence, in turn, the release of reactive oxygen and nitrogen species by microglia and astrocytes which may promote oxidative stress as well as enhance inflammatory pathways within the brain and such effects in morphology have also been found in patients with major depression [39]. 

### 3.3. Heart Rhythm Disturbances

In a recent meta-analysis, low heart beat rate observed in post-MI patients with depression has been associated with higher mortality, possibly because of the risk of enhancing the incidence of arrhythmias in this population [40]. Depression has also been found to be related with longer QT intervals, decreased baroreflex cardiac control and ventricular arrhythmias [41]. Lower heart rate variability (HRV) may indicate decreased parasympathetic tone, which allows sympathetic nerves to provide stimulation and possibly provoke ventricular arrhythmias.

### 3.4. Catecholamines

Veith et al. provided evidence that norepinephrine (NE) was significantly elevated into vascular and extravascular compartments of patients with depression compared to controls while both had similar plasma NE clearance rates, suggesting increased sympathetic nervous system activity [42]. Catecholamines cause vasoconstriction, arrhythmia, increased blood pressure, and platelet activation, all of which are contributing factors of cardiovascular instability [33]. 

### 3.5. Endothelial Dysfunction

The impaired endothelial function, usually appearing with aging, has been suggested as a contributing factor to the development, progression, and clinical manifestations of atherosclerosis [43]. Elevated symptoms of depression compared to nondepression in CHD patients have been associated with diminished flow-mediated dilation of the brachial artery, an index of endothelial function that is related to coronary vessel dysfunction [44]. Impaired nitric oxide (NO) function, a marker of endothelial dysfunction, has been reported in depressed patients [45]. NO is involved in several physiological activities including inflammation and vasodilation and impaired NO production can lead to vascular thrombosis and atherosclerosis [46]. 

## 4. Behavioral Mechanisms

It can be argued that patients with depression may exercise less and live a sedentary life, are less compliant with diet and drug treatment, and are more likely to consume alcohol and tobacco. As Gottlieb et al. reported, patients with depression may perceive that their quality of life (QoL) is lower and tend to underestimate their functional status [47]. However, objective assessment of physical functioning showed that depressed patients with heart failure (HF) had less exertion on exercise with lower respiratory quotient compared to nondepressed patients with HF. This finding could be of use when determining patients' functional class according to New York Heart Association (NYHA) criteria, as some depressed patients may report more severe physical status [48]. 

Similarly, Cheok et al. found that depressed patients at baseline in the Identifying Depression As a Comorbid Condition (IDACC) project [49] reported lower QoL as measured by different instruments: the Assessment of Quality of Life (AQoL) [50] and the Short Form-36 (SF-36) [51]. They found that depressive symptoms were related to younger age, female gender, unemployment, divorce or separation, and a lower educational level.

Compliance is another issue of great concern in patients with CVDs. Due to the fact that many patients are over 60 years old and suffering from other diseases as well, the number of medications may be increased with a number of them taken several times daily. In a study of elderly patients with HF, compliance rate was reported to be as low as 10% [52]. This strong association between noncompliance and depression was evident in a meta-analysis where nondepressed patients were three times more likely to adhere to their medical treatment compared to depressed patients [53]. 

Nevertheless, there is still much debate on whether depression itself, impaired cognition or associatedsequelaesuch as poor adherence to drug treatment, lack of exercise, sedentary life, and so on are responsible for adverse outcomes in CVD patients.

## 5. Prognosis

Around 20% of patients present with MDD and a larger portion may have subthreshold depressive symptoms [3, 6, 54, 55]. Depression is associated with increased morbidity, even greater than the impact that left ventricular function and ischemia have on CAD [56]. Another 15% to 23% of patients after an MI and in ACSs have depression and depression in this population increases mortality three times [57]. Lower QoL can be predicted in hospitalized ACS patients by the degree of depression [58]. 

In another study by Sherwood et al., it was found that severity of depressive symptoms but not use of antidepressants was related to increased likelihood of death or cardiovascular rehospitalization in HF patients [59]. In accordance with these findings, in a recent prospective study of 1006 patients with a mean follow-up period of 972 days, depression rather than antidepressants' use was independently associated with increased mortality in HF patients (hazard ratio HR: 1.34; 95% confidence interval: 1.08–1.68) [60]. 

Increased mortality, readmission rates, and cardiac events have also been associated with depression in patients undergoing CABG [61, 62]. Depressed HF patients experience a twofold increase in mortality risk [8], whereas depression worsens health status and increases hospitalization rates [9, 10, 63].

An interesting feature that has been brought to surface is the fact that onset of depression is related to morbidity and cardiac events, with patients presenting postcardiac event depression being at greater risk. Several studies showed that even after controlling for age, sex, education level, and left ventricular ejection fraction (LVEF) among others, depression is predictive of mortality and recurrent cardiovascular events [64–67]. According to an explanation offered bySpijkerman et al.[68] and Goodman et al. [69], new onset of depression may be associated with more severe state of cardiac disease or different risk factors compared to recurrent depression [70]. 

Faller et al., in a study of 231 HF patients with a median follow-up period of 986 days (IQR = 664–1120 days), found that major but not minor depression was associated with shorter survival rates (HR: 3.3; 95% CI = 1.8–6.1, Wald *χ* ^2^ = 15.2, *P* < 0.001 versus HR: 1.6; 95% CI = 0.8–3.1, Wald *χ* ^2^ = 1.6, *P* = 0.20) compared to nondepressed patients, regardless of sex [71]. 2847 community-dwelling people with CHD or congestive heart failure (CHF) (*n* = 450) and without any CVDs (*n* = 2397) were studied by Penninx et al. in order to investigate the effect of depression on cardiac mortality [72]. During 50 months of followup, depressed older people had significantly higher risk of mortality. Patients with CVDs and minor or major depression had 1.6 and 3.0 times greater relative risk (RR) of mortality compared to nondepressed CVD patients (95% CI = 1.0–2.7 and 95% CI = 1.1–7.8, resp.), with the risk being almost fourfold in the subgroup of CHD patients (RR = 3.9; 95% CI = 1.3–11.8). Similarly, non-CVD individuals with minor or major depression were more likely to die compared to non-CVD, non depressed individuals (RR = 1.5; 95% CI = 0.9–2.6 and RR = 3.9; 95% CI = 1.08–1.68, resp.).

## 6. Brief Considerations in Stroke Patients

According to the World Health Organization, it is estimated that 15 million people every year experience stroke with a third not surviving and another third having a permanent disability [43]. Stroke may have several detrimental physical and psychological effects both on patients and their caregivers as well. Among these effects, depression is commonly found in this population [73]. In a systematic review by Poynter et al., it was found that prevalence of depression in women was higher in 35 among 47 studies included; however, reasons for this finding are inconclusive [74]. The need for early recognition and diagnosis of poststroke depression (PSD) is emphasized in the literature [75]. In a study by Schmid et al. who followed a group of 367 stroke patients with and without depression for 12 weeks, it was found that dependence after stroke was independently associated with increased age, stroke severity but not baseline depression [76]. The authors also reported significant association between cognition and neurological impairment with functioning. PSD may lead to poor quality of life, worse prognosis, and increased mortality. Morris et al. and Ellis et al. found that PSD patients have higher mortality rates (3.4- and 1.88-fold risk, resp.) [10, 77]. Management strategies in PSD patients do not differ from those implemented in CVD patients with depression.

## 7. Treatment

According to the clinical guidelines (number 90 and 91, October 2009) issued by the National Institute for Health and Clinical Excellence (NICE) regarding treatment and management of depression in adults generally and adults with chronic physical health problems, respectively, there are several therapeutic approaches, varying from individual, computerized, or group cognitive behavioral therapy (CBT), interpersonal therapy (IPT), medications, or combinations of the above [78, 79]. 

### 7.1. Pharmaceutical Approaches

Several agents may be administered to patients suffering from depression. Some of the most frequently given include monoamine oxidase inhibitors (MAOIs), tricyclic antidepressants (TCAs), selective serotonin reuptake inhibitors (SSRIs), and serotonin norepinephrine reuptake inhibitors (SNRIs).

MAOIs and TCAs are generally avoided in patients with cardiovascular diseases since trials in antidepressants' use have shown adverse cardiac effects in patients without established heart disease [80–82]. In addition, they increase heart rate, cause orthostatic hypotension, impede cardiac conduction, and increase the risk of arrhythmias [83–85]. 

In one study, SSRIs have been shown to be safe for patients suffering from CVDs [86]. Nevertheless, the use of these drugs could still cause potential drug-to-drug interactions, since some SSRIs are metabolized by P450 liver enzymes, which metabolize drugs prescribed for treating certain CVDs. In another trial, investigators reported fluoxetine to be safe and effective in patients with stable CHD [87]. 

Sertraline was also found to be safe in patients hospitalized for AMI or unstable angina [88]. The authors reported that sertraline was superior to placebo mainly in a group of patients with severe recurrent depression. In a larger and more recent randomized, double-blind, placebo-controlled trial of sertraline that lasted 12 weeks (Sertraline Against Depression and Heart Disease in Chronic Heart Failure—SADHART-CHF), the drug was found safe; nevertheless, the administered doses (50 to 200 mg/day) did not significantly reduce depression, measured with HAM-D, compared to placebo [89]. 

The same scale was utilized in another study of citalopram by Fraguas et al. [90]. Apart from HAM-D, authors employed the MADRS and weekly visits by a psychiatrist. Generally, patients tolerated well this agent without any significant effects on left ventricular ejection fraction (LVEF), blood pressure, heart rate, or pulmonary function in this population of older people with heart failure. A concern about the suitability of HAM-D was raised by the authors who suggested that the MADRS may be more suitable for the evaluation of antidepressant effects in patients with HF and MDD.

Contrary to previous findings, clinical benefits derived in a Canadian study (Cardiac Randomized Evaluation of Antidepressant and Psychotherapy Efficacy—CREATE) evaluating citalopram alone and combined with interpersonal psychotherapy (IPT) [91]. Two hundred eighty-four patients with coronary artery disease (CAD) and major depression were allocated in four groups, receiving either IPT plus clinical management and citalopram (*n* = 67), IPT plus clinical management with placebo (*n* = 75), clinical management and citalopram (*n* = 75), or clinical management with placebo (*n* = 67). It was found that citalopram was effective and safe (effect size 0.33 in mean changes) after a 12-week period of drug use in this population that was required to have at least a score of 20 or higher in the HAM-D, meaning that the participants were more likely to be severely depressed. On the other hand, IPT did not exhibit any benefits over the usual clinical management which was a 20-minute, nonpsychotherapeutic intervention evaluating medication's adverse effects and depression symptoms.

Controlled-release paroxetine was studied in a pilot double blind, randomized, placebo-controlled study by Gottlieb et al. in a small population of patients with chronic heart failure [92]. At twelve weeks of followup, paroxetine-CR was associated with significant improvement in depression (69% versus 23%, *P* = 0.018) and improvements in psychological aspects of quality of life but not physical. In another trial, safety of paroxetine versus nortriptyline was examined [93]. By obtaining and analyzing baseline 24-hour ECG and 24-hour ECGs at the end of the second and sixth week of drug treatment, the authors concluded that paroxetine is a safer option in a population of patients with ischemic heart disease (IHD) and MDD, since nortriptyline had a greater vagolytic function, negatively influencing heart rate variability by prolonging R-R intervals.

Prevention of depression was studied in a double-blind randomized controlled trial which evaluated the efficacy of escitalopram in post-acute coronary syndromes (ACSs) patients [81]. There were no significant adverse events in the treatment group. A multivariate analysis revealed that the absence of escitalopram and a higher score in HAM-D scale were the only elements associated with development of depression in these patients who did not have clinical depression at baseline. Only two of the 120 patients treated with escitalopram developed depression versus ten in control group (*P* = 0.022).

In the Myocardial Infarction and Depression Intervention Trial (MIND-IT), a multicenter, double-blind, randomized controlled study, 91 post-MI patients (recruitment after 3 to 12 months post-MI) with depression were allocated to treatment with mirtazapine or placebo during a 24-week period [94]. Patients from both groups reported adverse effects such as fatigue, dizziness, headache, and appetite changes and there had been incidents of heart failure (*n* = 1), angina pectoris (*n* = 1), and atrial fibrillation (*n* = 1) in the intervention group. The number of hospitalizations was similar between the two groups (10 versus 8) and ECG characteristics such as PR and QRS duration and QTc interval did not show significant changes. Although scores in HAM-D did not show any substantial changes, statistically significant improvements were found in the depression subscale of the Symptom Check List 90 (dSCL-90) and the Clinical Global Impression (CGI) scale (*F* = 3.88, *P* = 0.02 and *F* = 3.87, *P* = 0.05, resp.).

Kerber at al. studied symptom remission in depressed patients with and without heart disease (HD), while administering different combinations of agents: bupropion sustained release plus escitalopram, venflaxine extended release plus mirtazapine, and escitalopram plus placebo [95]. In thissingle-blinded,prospective randomized trial, HD patients had similar remission rates (40% versus 38.2%, *P* = 0.556) and response rates (50% versus 52.1%, *P* = 0.805) and demonstrated fewer side effects at the 12th and 28th treatment week, indicating that heart disease may not influence the therapeutic effects of antidepressants and their use is safe. However, the number of HD patients included was small (*n* = 40) with a mean age of 42 years old and heart condition was self-reported.

 In another review of several studies in animals and humans [75], erythropoietin (EPO) was found to have beneficial effects on hippocampus-dependent memory and antidepressant-like effects that seem to be attributable to neuriological actions of EPO rather than increase of red cell mass. The authors conclude that, although studies on this area are still small scaled, EPO may be a promising add-on treatment for mood disorders.

In conclusion, it should be emphasized that a great number of studies performed on various drugs may be underpowered to efficiently address safety in CVD patients and some of them were of short duration. Thus, more studies are needed in this area.

### 7.2. Psychotherapy

Cognitive behavioural therapy (CBT) and interpersonal therapy (IPT) are two forms of psychotherapy that are employed in managing depression. CBT is based on the idea that individuals' thoughts cause their feelings and behaviour, not external things such as people, situations, or events. The benefit of this fact is that the way someone thinks can be changed in order to feel and act better even if the situation does not change. Emphasis is also placed on problem solving and on increasing the time and frequency of pleasurable activities [96].

IPT is a short-term supportive psychotherapy that focuses on the connection between interactions that people have and the development of psychiatric symptoms. There are several types of interventions that are commonly used in IPT: a focus on emotions; exploration of client's resistance to treatment; discussion of patterns in client's relationships and experiences; detailed past history; emphasis on client's current interpersonal experiences; exploration of the relationship between the client and the therapist; the identification of the client's wishes. IPT focuses on the ways in which a person's current relationships and social context cause or maintain symptoms instead of trying to explore the wider sources of the symptoms and aims at rapid symptom reduction and improved social adjustment.

The goals of IPT in the treatment of depression are to diagnose depression explicitly and educate the patient about depression, its causes, and the various treatments available for it; to identify the interpersonal context of depression as it relates to symptom development; to develop strategies for the patient to follow in coping with the depression. The targeted approach of IPT has demonstrated improvement for patients with problems ranging from mild situational depression to severe depression with a recent history of suicide attempts [97–101]. 

## 8. Future Research Directions

Our knowledge base concerning depression in patients with cardiac diseases is increasingly enriched by valuable studies on this area. However, not all pieces of the puzzle are there yet.

It is not quite clear, for example, whether there are any coincidental physiologic factors found in depressed patients that may or not affect the progression ofheartdisease per se. Also, another issue of concern may be the timing of diagnosing depression in patients with CVDs, as well as the use of a screening modality suitable for each heart condition. Also, it is hoped and widely expressed in the literature that advances in technology and genetics will allow us to image molecules that may be implicated in the pathophysiology of the brain activity in depressed patients and confirm possible areas in chromosomes that are hypothesized to be linked with depression.

Finally, it is encouraging to know that the National Institute of Mental Health (NIMH), acknowledging the importance of depression, has awarded researchers at the University of Pittsburgh School of Medicine a three-year, half a million dollars grant to develop an intervention strategy for simultaneously treating congestive heart failure and major depression. This future study will be designed to compare the impact of a “blended” depression/heart failure care management programs versus traditional heart failure care management program on cardiovascular morbidity and mortality, health-related quality of life, mood symptoms, health care costs, and a variety of other outcomes of interest, further enhancing our existing understanding.

## 9. Conclusions

It has been widely suggested in the literature that depression negatively affects patients with cardiovascular diseases. There are several pathophysiologic mechanisms linking depression and cardiac events as well as behavioral processes. Improvements in nursing and medical care have prolonged survival of this patient population; however, this beneficial outcome may have led to increased prevalence of depression. Since mortality rates in treated CHF patients remain extremely high, it might be as equally important to screen for depression and there are several valid and reliable screening tools that healthcare personnel, such as nurses, could easily employ in order to identify patients at greater risk. Identification is the first step to take. Consultations should follow by a multidisciplinary team, consisting of cardiologists, psychiatrists, and hospital or community nurses so as to carefully plan, execute, and evaluate drug treatment, medical interventions, and necessary lifestyle changes of cardiac patients who suffer from any form of depression.

## Figures and Tables

**Table 1:** ICD-10 (version 2010) and DSM-IV-TR criteria for depression.

Depressive episode according to ICD-10	Major depressive disorder according to DSM-IV-TR
Generally, the patient should present for at least 2 weeks with the following.	(1) At least one of the two main symptoms and five or more of the additional symptoms present during the same 2-week period
(a) Main symptoms	(a) Main symptoms
(i) Lowering of mood	(i) Depressed mood
(ii) Reduction of energy	(ii) Loss of interest or pleasure
(iii) Decrease in activity	(b) Additional symptoms
(b) Additional symptoms	Fatigue or loss of energy
(i) Reduced capacity for enjoyment, interest and concentration	Decreased ability to think or concentrate, indecisiveness
(ii) Marked tiredness after even minimum effort	Insomnia or hypersomnia
(iii) Disturbed sleep	Feelings of worthlessness or excessive, inappropriate guilt
(iv) Diminished appetite	Recurrent thoughts of death or recurrent suicidal ideation
(v) Reduced self-esteem and self-confidence	Psychomotor agitation or retardation
(vi) Ideas of guilt or worthlessness	Significant weight loss or weight gain
(c) Somatic symptoms (i) Loss of interest and pleasurable feelings (ii) Waking in the morning several hours before the usual time (iii) Depression is worst in the morning (iv) Marked psychomotor retardation, agitation (v) Loss of appetite (vi) Weight loss (vii) Loss of libido	(2) Symptoms do not meet the criteria for a mixed episode (3) Symptoms cause clinically significant distress or impairment in social, occupational, or other important areas (4) Symptoms are not due to direct physiological effects of a substance (i.e., drug or medication abuse) or medical condition (i.e., hypothyroidism) (5) Symptoms are not better accounted for by a bereavement (e.g., after the loss of a loved one); they persist for more than 2 months or are characterized by marked functional imparment, morbid preoccupation with worthlessness, suicidal ideation, psychotic symptoms, or psychomotor retardation.
Mild depressive episode:	
Two or three of the above symptoms are usually present. The patient is usually distressed by these but will probably be able to continue with most activities	
Moderate depressive episode:	
Four or more of the above symptoms are usually present and the patient is likely to have great difficulty in continuing with ordinary activities.	
Severe depressive episode without psychotic symptoms:	
An episode of depression in which several of the above symptoms are marked and distressing, typically loss of self-esteem and ideas of worthlessness or guilt.	
Suicidal thoughts and acts are common and a number of “somatic” symptoms are usually present.	

Adopted with permission from the American Psychiatric Association and the WHO (ID: 87085).
